# Conserved nematode signalling molecules elicit plant defenses and pathogen resistance

**DOI:** 10.1038/ncomms8795

**Published:** 2015-07-23

**Authors:** Patricia Manosalva, Murli Manohar, Stephan H. von Reuss, Shiyan Chen, Aline Koch, Fatma Kaplan, Andrea Choe, Robert J. Micikas, Xiaohong Wang, Karl-Heinz Kogel, Paul W. Sternberg, Valerie M. Williamson, Frank C. Schroeder, Daniel F. Klessig

**Affiliations:** 1 Boyce Thompson Institute for Plant Research, Ithaca, New York 14853, USA; 2 Department of Plant Pathology and Microbiology, University of California Riverside, Riverside, California 92521, USA; 3 Department of Plant Pathology and Plant-Microbe Biology, Cornell University, Ithaca, New York 14853, USA; 4 Research Centre for BioSystems, Land Use, and Nutrition, Justus Liebig University, Giessen D-35392, Germany; 5 Kaplan Schiller Research, LLC, Gainesville, Florida 32604, USA; 6 Howard Hughes Medical Institute and Biology Division, California Institute of Technology, Pasadena, California 91125, USA; 7 Robert W. Holley Center for Agriculture and Health, US Department of Agricultural Research Service, Ithaca, New York 14853, USA; 8 Department of Plant Pathology, University of California, Davis, California 95616, USA

## Abstract

Plant-defense responses are triggered by perception of conserved microbe-associated molecular patterns (MAMPs), for example, flagellin or peptidoglycan. However, it remained unknown whether plants can detect conserved molecular patterns derived from plant-parasitic animals, including nematodes. Here we show that several genera of plant-parasitic nematodes produce small molecules called ascarosides, an evolutionarily conserved family of nematode pheromones. Picomolar to micromolar concentrations of ascr#18, the major ascaroside in plant-parasitic nematodes, induce hallmark defense responses including the expression of genes associated with MAMP-triggered immunity, activation of mitogen-activated protein kinases, as well as salicylic acid- and jasmonic acid-mediated defense signalling pathways. Ascr#18 perception increases resistance in *Arabidopsis*, tomato, potato and barley to viral, bacterial, oomycete, fungal and nematode infections. These results indicate that plants recognize ascarosides as a conserved molecular signature of nematodes. Using small-molecule signals such as ascarosides to activate plant immune responses has potential utility to improve economic and environmental sustainability of agriculture.

## Abstract (web summary)

Plants are able to induce defense responses following recognition of certain pathogen derived molecules at the cell surface. Here, Manosalva *et al*. show that plants respond to ascarosides, a conserved class of nematode pheromones, providing the first example of plant recognition of an animal-derived signalling molecule.

## Graphical abstract

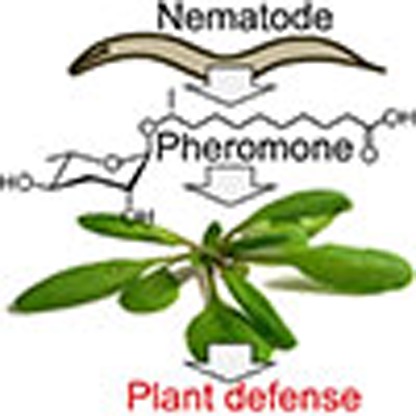

The recognition of specific molecular patterns has been shown to play a central role in the immune responses of plants and animals^1^,^2^. Both plants and animals possess pattern recognition receptors that serve to detect several different molecular signatures associated with specific classes of microbes. For example, *Arabidopsis* recognize bacteria using specific pattern recognition receptors for flagellin, lipopolysaccharide, peptidoglycan and other MAMPs^2^,^3^. MAMPs are often perceived at low concentrations and induce specific defense responses. Perception of MAMPs, has been shown to involve conserved signal-transduction mechanisms, including activation of mitogen-activated protein kinases (MAPKs), generation of reactive oxygen species and activation of salicylic acid (SA)- and jasmonic acid (JA)-signalling pathways^4^,^5^,^6^.

Nematodes are arguably the most numerous animals on earth^7^. They are ubiquitous in soil and parasitize most plants and animals. Plant-parasitic nematodes cause agricultural damage of more than $100 billion annually worldwide^8^. Several studies have shown that plants respond to plant-parasitic nematode inoculation by rapidly activating defense pathways similar to those induced by other pathogens in plants^9^,^10^,^11^. However, the nature of the nematode-derived signals that are perceived by plants has remained unknown. Interestingly, entomopathogenic nematodes, which do not parasitize plants, can also induce plant defenses, including expression of *PATHOGENESIS-RELATED PROTEIN-1* (*PR-1*) and increased catalase and peroxidase activity^12^. This suggests that recognition of a conserved nematode signature molecule could be responsible for the plant's defense response.

Recent work has shown that nematodes rely on an evolutionarily conserved family of signalling molecules, the ascarosides, to regulate development and social behaviours^13^,^14^,^15^,^16^,^17^,^18^,^19^,^20^,^21^. Ascarosides are glycosides of the dideoxysugar ascarylose that carry a fatty acid-derived lipophilic side chain and have been identified exclusively from nematodes. For example, in the model organisms *Caenorhabditis elegans*^22^ and *Pristionchus pacificus*,^23^, as well as in the insect-parasitic nematode *Heterorhabditis bacteriophora*,^20^ species-specific blends of ascarosides regulate entry into stress-resistant dispersal or infective larval stages. Different structural variants are associated with starkly different activity profiles, and biological activity is frequently observed at very low concentrations.^18^

More than 200 different ascaroside structures from over 20 different nematode species have been identified, demonstrating that ascarosides represent a highly conserved molecular signature of nematodes^18^. These findings suggested that plant and animal hosts of nematodes, as well as nematode-associated microorganisms, may have evolved the means to detect and respond to this ancient nematode signature. Notably, nematophagous fungi, which are natural predators of soil-dwelling nematodes, have recently been shown to initiate the formation of specialized trapping devices in response to ascarosides^24^,^25^.

In this study, we investigated whether ascarosides are perceived by plants and modulate plant-defense responses. We first characterized the ascaroside profiles of several agriculturally important species of plant-parasitic nematodes, including root-knot and cyst nematodes, which are the two most-damaging groups^26^. We then assessed whether ascarosides produced by plant-parasitic and other nematodes affect plant immunity. Ascarosides were found to trigger conserved defense responses in leaves and in roots, including the SA- and JA-mediated signalling pathways. Moreover, treatment with ascarosides enhances resistance to certain viral, bacterial, fungal and oomycete pathogens, as well as to root-knot and cyst nematodes in *Arabidopsis*.

## Results

### Plant-parasitic nematodes produce ascarosides

**Figure 1:**
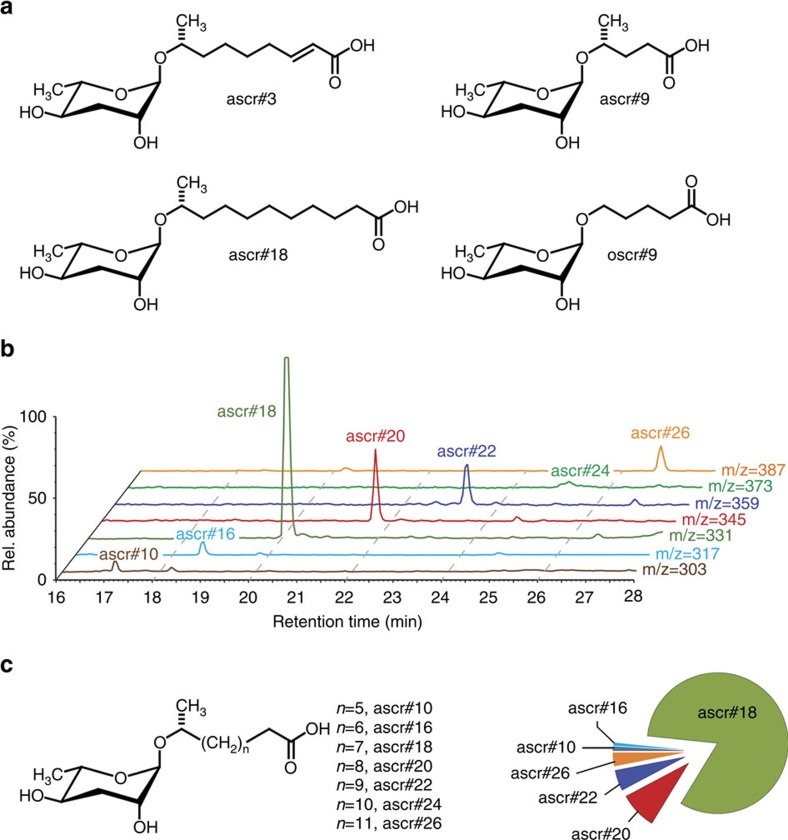
Identification of ascarosides from plant-parasitic nematodes. (**a**) Examples of ascarosides previously identified from *C. elegans* and other nematode species. (**b**) HPLC-MS analysis of *M. hapla* exo-metabolome samples, showing ion chromatograms scaled to 100% of the ascaroside peak corresponding to *m/z*=[M-H]^−^ for seven detected ascarosides. (**c**) Chemical structures of identified ascarosides and relative quantitative distribution as determined by HPLC-MS. For high-resolution MS data, see Supplementary Table 1. Quantitative ascaroside profiles of *M. incognita* and *M. javanica* are shown in Supplementary Fig. 1.

To investigate the possibility that plants respond to ascarosides, we first characterized the ascaroside profiles produced by several genera of plant-parasitic nematodes. We used media supernatant samples to analyze the excreted metabolome (‘*exo*-metabolome') of infective juveniles of three species of root-knot nematodes, *Meloidogyne incognita*, *M. javanica* and *M. hapla*, as well as cyst (*Heterodera glycines*) and lesion (*Pratylenchus brachyurus*) nematodes, using a recently developed sensitive and selective mass spectrometric (MS) screening method^27^ (Fig. 1). MS analysis of *exo*-metabolome samples revealed excretion of similar sets of ascarosides in all analyzed species. In *Meloidogyne* spp., ascr#18, a compound featuring an 11-carbon side chain, was most abundant, followed by compounds with longer carbon side chains (Fig. 1a–c; Supplementary Fig. 1 and Supplementary Table 1). Concentrations of ascr#18 in the analyzed *Meloidogyne* spp. culture media samples were variable and ranged from 5 nM to 100 nM. Analysis of *H. glycines* and *P. brachyurus* metabolome samples also revealed ascr#18, albeit in smaller amounts than in *Meloidogyne* spp. (Supplementary Table 1). *Exo*-metabolome samples of adult *M. hapla*, *H. glycines* and *P. brachyurus* contained trace amounts of ascr#18, whereas the other ascarosides found in infective juveniles could not be detected in adults. These results show that plant-parasitic nematodes, like most other previously analyzed nematode species^19^, produce ascarosides. Notably, the analyzed species from three genera of plant-parasitic nematodes all produce ascr#18 as the most abundant ascaroside. Ascr#18 had previously been identified as a minor component of the ascaroside profile produced by the model organism *C. elegans*^27^ and is also produced by entomopathogenic nematodes^19^,^20^.

### Ascr#18 induces defense responses and enhances resistance

**Figure 2:**
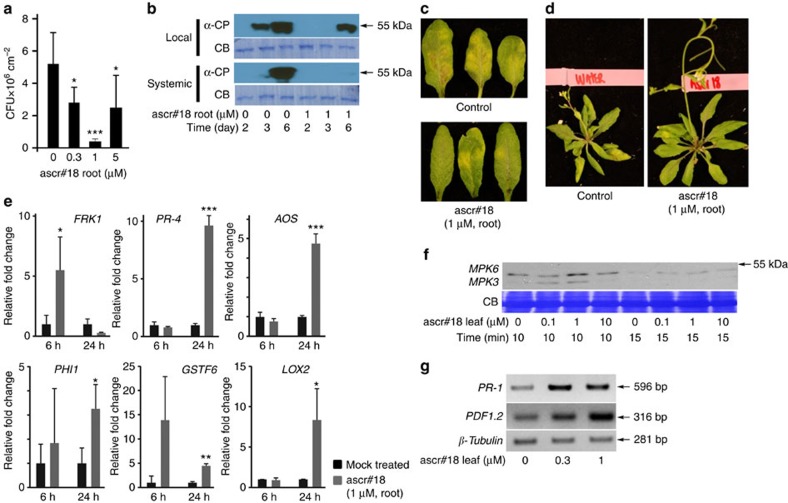
Ascr#18 enhanced pathogen resistance and activated defense responses in *Arabidopsis*. (**a**) Enhanced resistance to virulent *Pseudomonas syringae* pv. *tomato* (*Pst*) DC3000 after root pretreatment for 24 h with ascr#18. Bacterial growth was assayed at 3 d.p.i. Data are averages±s.d. (*n*≥3). (**b**) Quantification of TCV CP in inoculated (local) and uninoculated (systemic) leaves of plants root pretreated for 24 h with ascr#18. Leaves were harvested at 2, 3 and 6 d.p.i. for immunoblot analysis with the anti-CP antibody. Coomassie blue staining (CB) served as loading control. (**c**) TCV-inoculated (local) leaves photographed at 6 d.p.i. (**d**) TCV-infected plants photographed at 6 d.p.i. (**e**) Transcript levels as measured by qRT-PCR of defense-gene markers in leaves from plants root pretreated with ascr#18 (1 μM). Gene-transcript levels were determined at 6 h.p.t. and 24 h.p.t. Data are average±s.d. (*n*=3). (**f**) Activation of MAPKs MPK3 and MPK6 in *Arabidopsis* 10 and 15 min after leaf pretreatment with ascr#18. CB served as loading control. (**g**) Induction of SA and JA marker genes *PR-1* and *PDF1.2*, respectively, after syringe infiltration of leaves with ascr#18, as measured by qRT-PCR. *β-tubulin* was used as internal control. *P≤0.05; **P≤0.005; ***P≤0.0005, two-tailed *t*-test.

On the basis of the finding that ascr#18 is produced by all analyzed species of plant-parasitic nematodes, we asked whether this ascaroside is perceived by plants and affects plant-defense responses to diverse pathogens. Because ascarosides as the nematode signalling molecules may have direct effects on nematode pathogens that could confound detection of plant responses to ascr#18, we began by testing the effect of ascr#18 on defense responses of *Arabidopsis* to a bacterial (*Pseudomonas syringae* pv. tomato) and a viral pathogen (Turnip Crinkle Virus—TCV; Fig. 2). Since plants would naturally encounter nematodes via their roots, *Arabidopsis* roots were partially immersed in water containing different concentrations of ascr#18 for 24 h before leaves were inoculated with the pathogens. Root treatment with 1 μM ascr#18 reduced growth of virulent *P. syringae*, whereas a higher ascr#18 concentration (5 μM) was less effective (Fig. 2a). Root treatment with ascr#18 at 1 μM also enhanced resistance to virulent TCV (Fig. 2b–d, Supplementary Fig. 2.). Viral replication, as measured by the amount of viral coat protein (CP) in leaves (Fig. 2b), was reduced in both inoculated and distal leaves. Moreover, systemic spread of the virus was nearly abolished with only a trace of CP present in distal leaves of ascr#18-pretreated plants. Disease symptoms, including development of chlorosis on the inoculated leaves (Fig. 2c), leaf curling/crinkling and suppression of inflorescence development (Fig. 2d), were also reduced in ascr#18-pretreated plants.

To further characterize *Arabidopsis*' response to ascr#18, we monitored expression of MAMP-triggered immunity (MTI) markers and defense-related genes in leaves at different time points after root treatment with ascr#18. MAPKs and calcium-dependent protein kinase are key components of signalling pathways that regulate recognition of MAMPs by plants^5^,^28^. We measured induction of the MAPK-related *Flg22*-*INDUCED RECEPTOR KINASE1* (*FRK1*) and the calcium-dependent protein kinase-related *PHOSPHATE-INDUCED1* (*PHI1*) MTI marker genes^28^. Pretreatment of roots with ascr#18 induced increased transcript levels in the leaves of *FRK1* at 6 h post treatment (h.p.t.) and *PHI1* at 24 h.p.t. (Fig. 2e). In addition, expression of representative biosynthetic or responsive genes for the two major hormones mediating plant immunity, SA and JA, was also affected. Root pretreatment with ascr#18 increased expression of the SA-responsive genes *PATHOGENESIS-RELATED-4* (*PR-4)* and *GLUTATHIONE S-TRANSFERASEF6* (GSTF6), and the JA-biosynthetic genes *LIPOXYGENASE2* (*LOX2*) and *ALLENE OXIDE SYNTHASE* (*AOS*) in leaves (Fig. 2e)^6^.

Because JA and SA signalling interact with the ethylene (ET) and auxin signalling pathways^6^, we additionally monitored changes in the expression of five genes associated with ET signalling, as well as five genes associated with auxin signalling in response to root treatment with 1 μM ascr#18 at 24 h.p.t. Of the 10 tested genes, only expression of *SAUR-LIKE AUXIN RESPONSE PROTEIN34 (SAUR34)* was enhanced (Supplementary Fig. 3), suggesting that ascr#18 treatment does not strongly affect auxin- or ET-regulated defense signalling.

Next we assessed whether plants respond to ascr#18 via their leaves. Leaf infiltration of ascr#18 induced activation of the MAPKs, MPK3 and MPK6, which are early markers for the development of MTI (Fig. 2f, Supplementary Fig. 4)^5^. MAPK activation was monitored via immunoblot analysis using an antibody that detects phosphorylation of the pTE-pY motif^29^. We observed a transient increase in the phosphorylation of both MAPKs within 10 min after leaf infiltration with 1 μM ascr#18. In addition, transcripts for the prototypic SA-responsive marker *PR-1* and prototypic JA-responsive *PDF1.2* genes^6^ were elevated at 24 h.p.t. after infiltration with 0.3 μM or 1 μM ascr#18 (Fig. 2g, Supplementary Fig. 5). Taken together, our results show that in *Arabidopsis*, local and systemic defenses are activated in response to ascr#18 application to leaves or roots.

### Ascr#18 enhances resistance in dicot and monocot crop plants

**Figure 3:**
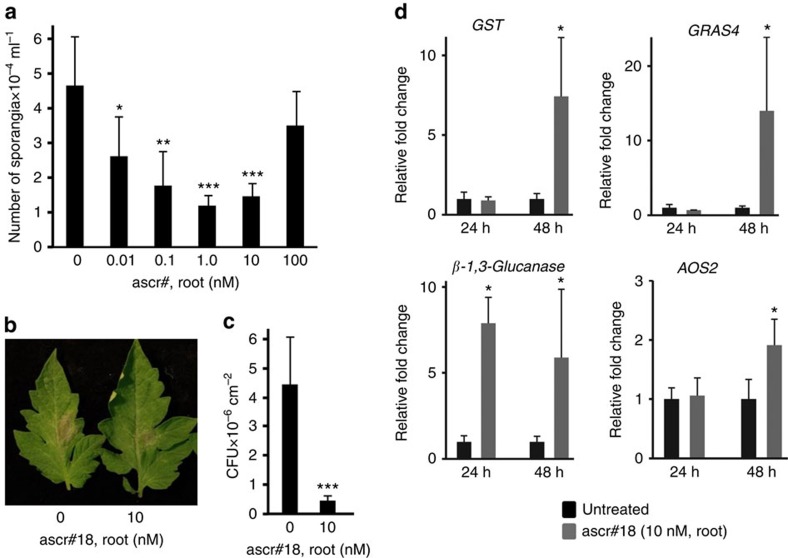
Ascr#18 enhanced pathogen resistance and activated defense responses in tomato. (**a**) Dose dependence of enhanced resistance to *Phytophthora infestans* (US22) in leaves of tomato cv. M82 root pretreated with ascr#18 for 48 h before inoculation. Sporangia numbers were count at 6 d.p.i. to assess disease severity. Data are average±s.d. (*n*=16). (**b**) Blighted area caused by *P. infestans* in tomato cv. M82 leaves photographed at 6 d.p.i. (**c**) Enhanced resistance to *Pst DC3000* in tomato cv. M82 root pretreated with ascr#18 for 48 h. Bacterial growth was assayed at 4 d.p.i. Data are average±s.d. (*n*=6). (**d**) Induction of defense-response genes in tomato leaves 24 and 48 h after root pretreatment with ascr#18 (see Fig. 2e legend for details). Data are average±s.d. (*n*=3). *P≤0.05; **P≤0.005; ***P≤0.0005, two-tailed *t*-test.

To determine whether ascaroside perception is conserved across the plant kingdom and to test for effects on resistance to eukaryotic pathogens, we measured the effect of ascr#18 on defense responses and pathogen resistance of the dicots tomato (*Solanum lycopersicum*) and potato (*Solanum tuberosum*), as well as the monocot barley (*Hordeum vulgare*). In tomato, pretreatment of roots with 1 nM and 10 nM of ascr#18 for 48 h prior to inoculation provided strong protection against the oomycete pathogen *Phytophthora infestans* as indicated by the reduction in sporangia number and lesion size (Fig. 3a,b, Supplementary Fig. 6). Similar to the effect of ascr#18 on resistance of *Arabidopsis* to *Pst*, protection against *P. infestans* in tomato decreased at higher concentrations of ascr#18. However, maximal protection was observed at ascr#18 concentrations (1–10 nM, Fig. 3a), much lower than in the case of *Arabidopsis* (1 μM, Fig. 2a). Root pretreatment with ascr#18 at 10 nM also reduced the growth of *Pst* in tomato leaves (Fig. 3c), whereas higher concentrations were much less effective (Supplementary Fig. 7). Root treatment of tomato with ascr#18 induced the accumulation of transcripts in the leaves for (i) the transcription factor *GRAS4*, a known marker of MTI linked to abiotic- and biotic-stress responses in tomato^30^, (ii) the SA-responsive genes *GST* and *β-1,3-GLUCANASE* and (iii) the JA-biosynthesis gene *AOS2* (Fig. 3d)^6^. Transcript levels for all four genes were significantly elevated at 48 h.p.t., with *β-1,3-GLUCANASE* also exhibiting enhanced levels at 24 h.p.t. Pretreatment with 10 nM ascr#18 either by root bathing or foliar spray 48 h prior to inoculation also provided protection against virulent *P. infestans* in three cultivars of potato (Supplementary Fig. 8).

**Figure 4:**
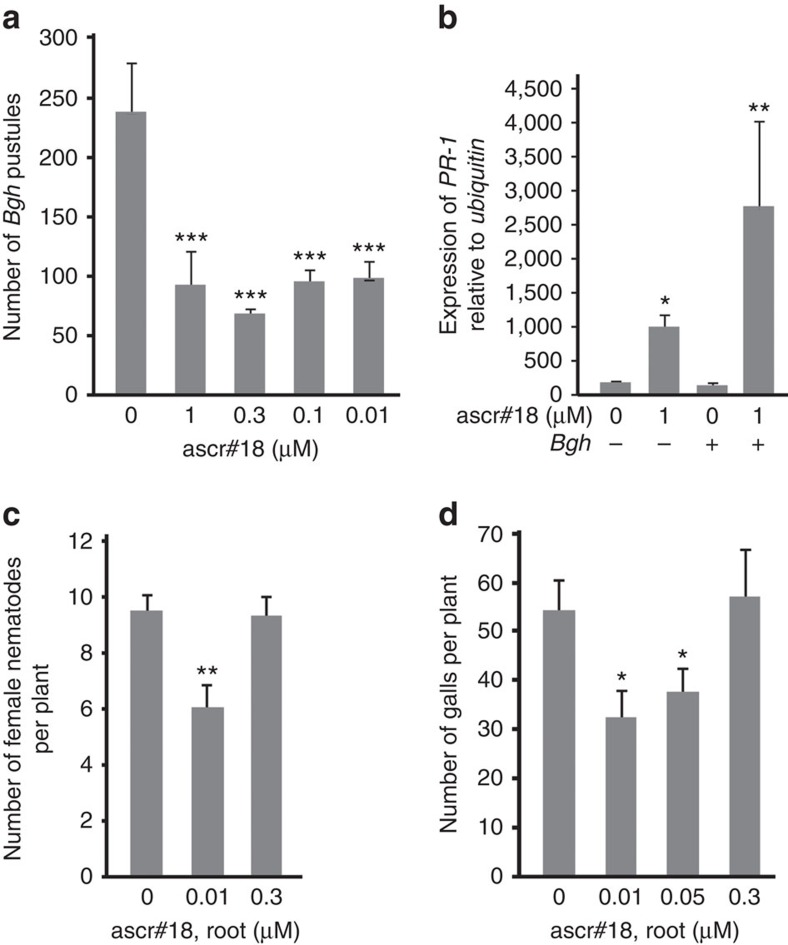
Effects of ascr#18 on resistance to a fungal pathogen in barley and to two species of nematodes in *Arabidopsis*. (**a**) Effect of leaf pretreatment with ascr#18 on resistance of barley to *Blumeria graminis* f. sp. *hordei* (*Bgh*). Leaves were sprayed with ascr#18 solutions 48 h before inoculation. *Bgh* pustules were counted at 7 d.p.i. Data are average±s.d. (*n* =10). (**b**) Induction of *PR-1* expression by leaf pretreatment with ascr#18. Plants were inoculated with *Bgh* 48 h post pretreatment and leaves were collected at 16 h.p.i. for qRT-PCR analysis. Data are average±s.d. (*n*=3). (**c**) Effect of ascr#18 on *Arabidopsis* susceptibility to sugar-beet cyst nematode (*H. schachtii*). Ten days-old *Arabidopsis* seedlings were pretreated with buffer or 0.01 and 0.3 μM of ascr#18 for 48 h before inoculation with about 200 freshly hatched and surface-sterilized juveniles per seedlings. The numbers of females were counted 4 weeks after inoculation. (**d**) Effect of ascr#18 on *Arabidopsis* susceptibility to root-knot nematode (*M. incognita*). Ten day-old *Arabidopsis* seedlings were pretreated with the indicated ascr#18 concentrations for 48 h before inoculation with ∼300 freshly hatched and surface-sterilized juveniles per seedlings. The numbers of galls were counted under microscope 6 weeks after inoculation. Data are average±s.d. (*n*=5). (*P≤0.05; **P≤0.005; ***P≤0.0005, two-tailed *t*-test).

To test whether ascr#18 activates defense responses in monocots, leaves of barley (*Hordeum vulgare*) were sprayed with ascr#18 48 h prior to inoculation with the virulent fungal pathogen *Blumeria graminis* f. sp. *hordei* (*Bgh*). Pretreatment with a wide range of ascr#18 concentrations (0.01–1 μM) increased resistance to *Bgh*, based on the reduced numbers of pustules formed on the leaves (Fig. 4a). This pretreatment also induced *PR-1* transcript accumulation; even higher levels of *PR-1* expression were observed in ascr#18-pretreated leaves that were subsequently inoculated with *Bgh*, suggesting a priming effect of ascr#18 in barley (Fig. 4b). Taken together, our results show that ascr#18 is perceived by monocots and dicots and induces defense responses that enhance resistance against four major classes of pathogens.

### Ascr#18 enhances resistance of *Arabidopsis* to nematodes

Next, we tested whether root exposure to ascr#18 affected infection of *Arabidopsis* by plant-parasitic nematodes. Using a range of ascr#18 concentrations, we found that pretreatment of roots with 10 nM ascr#18 significantly reduced infection of *Arabidopsis* with cyst (*H. schachtii*) and root-knot (*M. incognita*) nematodes (Fig. 4c,d), whereas higher concentrations of ascr#18 were not effective. Next we assessed whether treatment of *Arabidopsis* with nanomolar concentrations of ascr#18 affected root expression of the defense-related genes *PHI1*, *FRK1* and *WRKY53*, which encodes an immune-modulating transcription factor. We found that all three genes were induced within 6 h of exposure to 10 or 50 nM ascr#18, whereas exposure to a higher concentration (300 nM) had no effect or reduced expression (Supplementary Fig. 9).

### Plant responses to other ascarosides

In order to assess whether plants respond to ascarosides as a compound class, we tested three additional ascarosides whose structures differ from that of ascr#18 in several different ways, for their ability to induce defense responses. For this we selected ascr#3, an ascaroside that includes a conjugated double bond in the side chain, as well as ascr#9 and oscr#9, which both have a much shorter side chain (5 carbon) than ascr#18 (11 carbons, Fig. 1a)^17^. Similar to ascr#18, leaf infiltration of *Arabidopsis* with either ascr#3 or ascr#9 induced expression of the prototypic SA-responsive *PR-1* and JA-responsive *PDF1.2* genes, whereas oscr#9 showed no effect (Supplementary Table 2). Moreover, root pretreatment of tomato with ascr#9, like ascr#18, enhanced resistance to *P. infestans*, whereas neither ascr#3 nor oscr#9 affected resistance at the concentration tested. These results show that plants respond to structurally diverse ascarosides, but that responses vary in a structure- and species-dependent manner. Notably, oscr#9, whose structures differ from that of ascr#9 only in the position of the attachment of the side chain to ascarylose, was inactive in all assays, whereas ascr#9 was active. The possibility that the observed increases in pathogen resistance were due to anti-bacterial or anti-fungal activity of ascarosides is unlikely since a previous study showed that *C. elegans* metabolome samples containing micromolar to millimolar concentrations of ascarosides have no anti-microbial activity^31^.

## Discussion

Our work shows that ascarosides, a class of small molecules specific to nematodes, are perceived by plants as a conserved foreign molecular signature. Analogous to the effects of MAMPs such as flagellin^2^,^28^,^32^, perception of ascarosides by plants as ‘nematode-associated molecular patterns' leads to activation of conserved immune responses, resulting in enhanced resistance to a broad-spectrum of pathogens and pests. Plants respond to ascr#18, the most abundant ascaroside in plant-parasitic nematodes, at very low concentrations, similar to those required for perception of MAMPs^2^,^33^, suggesting that plants have evolved specific receptor(s) for ascaroside perception. Similar to bacterial MAMPs, sensitivity to ascarosides varied between plant species: in tomato, potato and barley 10 nM ascr#18 strongly induced defense-gene expression and enhanced resistance, whereas *Arabidopsis* required much higher concentrations. Notably, in both *Arabidopsis* and tomato, efficacy decreased at the highest tested ascr#18 concentrations. For example, in tomato ascr#18 concentrations of 1–10 nM provided maximal resistance to *P. infestans*, whereas enhancement of resistance was reduced or lost at 100 nM.

A similar decrease of activity at higher concentrations has been observed for ascaroside-mediated phenotypes in nematodes. For example, attraction of male *C. elegans* to hermaphrodite-produced ascarosides, as well as aggregation of *C. elegans* hermaphrodites in response to indole ascarosides is maximal at picomolar concentrations, but decreases markedly at higher concentrations^15^,^34^. The cause of the observed decrease in activity at higher ascaroside concentrations is not known. However, it has been suggested that high concentrations of small-molecule ligands can result in unproductive engagement of receptors, for example, by interfering with formation of receptor dimers^35^, which have been shown to be required for ascaroside perception in *C. elegans*^36^.

The ascr#18 concentration providing best protection against root infection with the root-knot and cyst nematodes (10 nM) in *Arabidopsis* was much lower than the concentration needed for best protection against leaf infection with bacterial and viral pathogens tested (1 μM). This may reflect higher expression of ascaroside receptors in the roots or differences in the distances between the sites of infection from the site of ascr#18 application or perception. In addition, the observed reduction of nematode infection levels may be due to direct effects of ascr#18 on these nematode species, which remain to be investigated. However, the induction of defense-associated genes in the roots at the effective ascr#18 concentration suggest that the increased resistance is, at least in part, mediated by enhanced immunity. Nanomolar concentrations of ascr#18 are representative of those found in culture media samples of the analyzed plant-parasitic nematode species, supporting the biological significance of ascr#18 in plant-nematode interactions.

The systemic induction of defense genes and pathogen resistance in leaves following root application suggests that ascr#18 itself (or a metabolite of ascr#18) moves from the roots to the leaves, and/or that ascr#18 induces synthesis of a mobile signal in the roots that then travels to the leaves to activate immune responses. We have not been able to detect ascr#18 in the leaf tissue of root-treated plants, but this could be due to limited uptake of the already very low concentrations of ascr#18 used in the assays. Although plants primarily encounter ascarosides via their roots, leaf infiltration and foliar spraying with low ascr#18 concentrations were also effective at inducing the defense responses. This finding suggests that ascaroside receptors are also present in leaf tissue. Further characterization of plant responses to ascarosides and identification of cognate receptors may reveal how perception of nematode-associated molecular patterns, MAMPs and other molecular patterns converges on triggering plant immunity. The use of small-molecule signals, such as ascarosides, to activate plant immune responses could help improve the economic and environmental sustainability of agriculture.

## Methods

### Worm sample preparation

Nematode eggs (*M. hapla* strain VW9, *M. incognita* VW6, *M. javanica* VW4 and *P. brachyurus*) were extracted from greenhouse-grown tomato plants, and surface-sterile juveniles were prepared as described in Branch *et al*.^37^. *H. glycines* was raised on greenhouse-grown soybean. Batches of ∼30,000–100,000 freshly hatched juveniles were incubated for 24 h in 8 ml sterile water with rotation. After brief centrifugation, the supernatant was filtered through a 22-μm filter then frozen. Filtered supernatants and worm pellets were lyophilized, each extracted with 2 × 5 ml methanol and filtered over cotton wool. Extracts were concentrated in vacuum and resulting residues were resuspended in 150 μl methanol and filtered prior to MS analysis.

### MS analysis

Low-resolution HPLC-MS and HPLC-MS/MS was performed using an Agilent 1100 Series HPLC system equipped with an Agilent Eclipse XDB-C18 column (9.4 × 250 mm, 5 μm particle diameter) and connected via a diode array detector to a Quattro II mass spectrometer (Micromass/Waters) using a 10:1 split. A 0.1% acetic acid—acetonitrile solvent gradient was used at a flow rate of 3.6 ml min^−1^, starting with an acetonitrile content of 5% for 5 min which was increased to 100% over a period of 40 min. Nematode metabolite extracts (prepared as described above) and synthetic samples (see Supplementary Methods and von Reuss *et al*.^27^) were analyzed by HPLC-ESI-MS in negative and positive ion modes using a capillary voltage of 3.5 kV and a cone voltage of −40 and +20 V respectively. HPLC-MS/MS screening for precursor ions of *m/z*=73.0 (negative mode) and neutral loss of 130.0 (positive mode) was performed using argon as collision gas at 2.1 mtorr and 30 eV. To confirm elemental composition of the identified compounds, samples were additionally analyzed by high-resolution mass spectrometry, using a Waters nanoACQUITY UPLC System equipped with a Waters Acquity UPLC HSS C-18 column (2.1 × 100 mm, 1.8 μm particle diameter) connected to a Xevo G2 QTof Mass Spectrometer. MassLynx software was used for MS data acquisition and processing.

### Plant material and growth conditions

Unless otherwise stated, *Arabidopsis thaliana* ecotype Col-0, tomato (*Solanum lycopersicum)* cultivar M82 and potato (*S. tuberosum)* cultivars Desiree, Eva and Yukon Gold plants were grown in a growth chamber under 16-h light/8-h dark (22 °C) regime and 70% relative humidity. Tomato and potato plants were grown in growth chambers for 3 weeks and then transferred to greenhouse conditions until they were used.

### Ascaroside treatments

Ascarosides were dissolved in ethanol to prepare millimolar stock solutions. Final aqueous ascaroside dilutions were prepared fresh on the day of the experiment. Control solutions contained equal amounts of ethanol (<0.1% for most experiments). For root treatment, plant pots were placed in a tray containing control solution or water supplemented with ascr#18. For leaf treatment, three leaves of 3.5-weeks-old *Arabidopsis* plants were syringe infiltrated with buffer (Bis-Tris pH 6.5) supplemented with an ethanolic solution of ascr#18 or buffer containing an equivalent amount of ethanol. For spray treatment, leaves were sprayed with a aqueous 0.02% Tween-20 solution to which either an ethanol solution of ascr#18 or ethanol (control) had been added.

### MAPK activation/phosphorylation

Leaf discs were collected from ascr#18-treated and mock-treated plants at different time points. Leaf tissue was frozen in liquid nitrogen and ground to a fine powder before adding 50 μl 4 × SDS protein sample buffer. Total cellular proteins were separated by electrophoresis in 8% SDS-PAGE. MAPK activation was detected by immunoblot analyses using 1:1,000 dilutions of polyclonal primary antibodies against phosphor-p44/42 MAPK (Cat No.# Antibody 9101, Cell Signaling Technology), which detects phosphorylation of the pTE-pY motif responsible for activation.

### Plant infection assays

For bacterial growth assays, 3 leaves of 3.5-weeks-old ascr#18-pretreated and mock-pretreated *Arabidopsis* plants were syringe infiltrated with a suspension of virulent *Pst* DC3000 in 10 mM MgCl_2_ at a density of 1 × 10^5^ colony-forming units (c.f.u.) ml^−1^ or with 10 mM MgCl_2_ only as control. Bacterial count was done 3 days post inoculation (d.p.i.) in *Arabidopsis* as described in Tian *et al*.^38^. Briefly, 3 leaf discs with a diameter of 0.7 cm were collected from 3 plants and placed into a single tube, serving as 1 replicate. Following bacteria recovery using 1 ml of 10-mM MgCl_2_, serial dilutions and plating were performed. In all, 20 μl from each tube were added to the wells of a microtitre plate containing 180 μl of 10-mM MgCl_2_, and serial 10-fold dilutions were prepared with a multi-channel pipette. Drops of 5 μl from each dilution were spotted onto a 150-mm Petri plate of LB containing rifampicin, and the plates were incubated at 28 °C. Bacterial counts were performed 48 h after incubation. TCV inoculation of 4-week-old plants was performed as described in Kang *et al*.^39^. In brief, *in vitro* transcripts of a cDNA clone were used at a final concentration of 35 ng μl^−1^; 2 μl were applied for each of the three *Arabidopsis* leaves. TCV CP was quantified using immunoblot analyses using 1:10,000 dilutions of anti-CP antibody, raised in a rabbit, and 1:10,000 secondary anti-rabbit antibodies (Sigma-Aldrich). For tomato and potato, 6-week-old plants were pretreated with ascr#18 or control solution 48 h before inoculation. Plants were infected with a virulent strain of *Phytophthora infestans* (US22) using a detached-leaflet assay as described in Manosalva *et al*.^40^. Briefly, the abaxial leaflet surface was inoculated by dropping 20 μl of sporangia suspension (4,000 sporangia ml^−1^). Sporangia suspension was incubated for 3 h at 4 °C for zoospore release before leaflet inoculation. The inoculated leaflets were kept in Petri dishes containing water agar and incubated at 15 °C. Blighted area was measured at 5 d.p.i. and sporangia numbers were counted at 7 d.p.i. For *Pst* bacterial growth assays in tomato, ascr#18-pretreated and mock-treated plants were vacuum-infiltrated with a suspension of virulent *Pst* DC3000 in 10-mM MgCl_2_ containing 0.02% Silwet L-77 at a density of 1 × 10^5^ c.f.u. ml^−1^. Plants were dipped upside down in 4 l of bacterial suspension and a vacuum was applied for 1 or 2 min followed by a slow release to infiltrate the leaves uniformly. Plants were then incubated in a growth chamber with 16-h illumination and 60% humidity at 22 °C. Bacterial count was done 4 d.p.i.

For barley cv. Golden Promise, 7 day-old seedlings were pretreated by spraying with aqueous solutions containing different concentrations of ascr#18. Inoculation with *Bgh* was done 48 h.p.t. using a detached-leaf assay. Briefly, 10 leaves from 5 different barley plants per pretreatment were cut and transferred to Petri dishes containing 1% water agar. Leaves were then inoculated with *Bgh*. Pustules were counted 7 d.p.i.

For *Arabidopsis* assays of nematode infection, *H. schachtii* and *M. incognita* were propagated and hatched as described previously^41^. Briefly, *Heterodera schachtii* and *Meloidogyne incognita* were propagated on greenhouse-grown cabbage (*Brassica oleracea* cv. All Season) and tomato (*Solanum lycopersicum* cv. Tiny Tim), respectively. To isolate *H. schachtii* eggs, nematode cysts were gently crushed in a glass homogenizer, and the eggs were collected and rinsed in water on to a 25-μm sieve. *M. incognita* eggs were isolated from egg masses on tomato roots with 0.5% sodium hypochlorite and rinsed with water on a 25-μm sieve. Collected nematode eggs were treated in a solution of 0.02% sodium azide for 20 min, and then hatched over water containing 1.5 mg ml^−1^ gentamycin sulfate and 0.05 mg ml^−1^ nystatin at room temperature on a Baermann pan for 3 days. Hatched second-stage juveniles (J2) were collected, surface sterilized with an aqueous solution of mercuric chloride (0.004%) and sodium azide (0.004%) for 10 min, and rinsed three times with sterile distilled water. Surface-sterilized J2 were resuspended in 0.1% agarose at a concentration of 10 J2 larvae per 10 μl and used for *Arabidopsis* inoculation*. Arabidopsis thaliana* ecotype Col-0 seeds were surface sterilized and planted in six-well plates with Knop medium containing 2% sucrose^42^. Plants were grown at 24 °C under 16-h light/8-h dark conditions. Two ml of various concentrations of ascr#18 or control solution were added to each well containing 10-day-old seedlings. After 48 h of pretreatment, the solution was removed and ∼200 freshly hatched and surface-sterilized J2 of *H. schachtii* or ∼300 freshly hatched and surface-sterilized J2 of *M. incognita* were inoculated on the roots of each seedling. Twenty-four seedlings were included for each treatment. Nematode females for *H. schachtii* were counted under microscope 4 weeks after inoculation. Galls for *M. incognita* were counted under microscope 6 weeks after inoculation.

### RNA analyses

Unless stated otherwise, three biological replicates were performed. For each replicate, total RNA from *Arabidopsis* leaves was isolated from a pool of one leaf from each of three plants. For tomato, RNA was isolated from six leaf discs per leaf per plant. Total RNA was isolated using Qiagene RNeasy Plant Mini Kit (Qiagen) according to the manufacturer's instructions. DNAse treatment was done using DNA-*free* Kit (Ambion) following the manufacturer's instructions. First-strand cDNA was synthesized from 1 μg of RNA using SuperScript II (Life Technologies) and amplified using gene-specific primers (Supplementary Table 3). Control reactions to normalize RT-PCR amplifications were run with the primers for constitutively-expressed *Arabidopsis β-tubulin* and for tomato translation *elongation factor 1α* (*EF1α*) gene. For quantitative real-time PCR (qRT-PCR), transcripts were amplified using IQ SYBR Green Supermix (Bio-Rad) from 5 μl of 10 × -diluted cDNA in a total 20 μl reaction using 1 μl of 10 μM gene-specific primers (Supplementary Table 3). Reactions were done using a CFX96 Touch Biorad Real-Time PCR System (Bio-Rad). The PCR conditions were 95 °C for 3 min (initial denaturation) followed by 40 cycles of amplification (95 °C for 15 s, 60 °C for 60 s), followed by generation of a dissociation curve. Three technical replicates were performed for each of the three biological replicates. The transcript level of defense-response genes in *Arabidopsis* and tomato are shown as fold change relative to mock-treated plants. The relative fold change was calculated according to the 2^−ΔΔCt^ method^43^. *Ubiquitin* (*Arabidopsis*) and *actin* (tomato) were used as endogenous reference genes. The paired *t*-test with an α-level of 0.05 was used to compare transcript level in the ascr#18-treated versus the mock-treated samples.

For barley analyses, RNA extraction from infected barley leaves was performed with TRIzol (Invitrogen) following the manufacturer's instructions. Freshly extracted mRNA was used for cDNA synthesis using QuantiTect Reverse-Transcription kit (Qiagen). cDNA was stored at −20 °C. For qRT-PCR, 50 ng of cDNA was used as template in the Applied Biosystems 7500 FAST Real-Time PCR system. Amplifications were performed in 7.5 μl of SYBER green JumpStart Taq ReadyMix (Sigma-Aldrich) with 0.5 pmol oligonucleotides. Relative quantification of the transcript abundance for barley genes were done using the 2^ΔCt^ method as described^44^. In barley, *Ubiquitin* was used as an endogenous reference gene.

For the RNA analyses in roots, *Arabidopsis* roots were collected from 20–30 seedlings for each treatment and mRNA was isolated using the Dynabeads mRNA DIRECT Kit (Life Technologies). DNA contamination was removed by treatment with DNase I (Life Technologies). First-strand cDNA was synthesized from 50 ng of mRNA using ProtoScript II Reverse Transcriptase (NEB). The qRT-PCR assay was carried out in an iCycler iQ Real-Time PCR Detection System (Bio-Rad) and transcripts were amplified using iTaq Universal SYBR Green Supermix (Bio-Rad) from 1 μl of 20 × -diluted cDNA in a total 20 μl reaction using 1 μl of each 10 μM gene-specific primer (Supplementary Table 3). All assays consisted of three technical replicates for each RNA sample. Data was analyzed using the iCycler iQ Real-Time PCR Detection System Software version 3.0a (Bio-Rad). The *Arabidopsis UFP* gene (AT4G01000) was used as an endogenous reference gene. PCR was started with an activation and DNA denaturation step (95 °C for 3 min), then followed by 40 cycles of 95 °C for 20 s and 60 °C for 40 s. The relative fold change was calculated according to the 2^−ΔΔCt^ method^43^.

## Additional information

**How to cite this article:** Manosalva, P. *et al*. Conserved nematode signalling molecules elicit plant defenses and pathogen resistance. *Nat. Commun.* 6:7795 doi: 10.1038/ncomms8795 (2015).

## Supplementary Material

Supplementary Information Supplementary Figures 1-9, Supplementary Tables 1-3, Supplementary Methods and Supplementary References
