# Supplementary material for: Conserved nematode signalling molecules elicit plant defenses and pathogen resistance
Source: Nat Commun. 2015 Jul 23;6:7795. doi: 10.1038/ncomms8795 (PMC4525156; doi:10.1038/ncomms8795)
Supplement: Supplementary Information — Supplementary Figures 1-9, Supplementary Tables 1-3, Supplementary Methods and Supplementary References [file ncomms8795-s1.pdf]

Supplementary Information

Supplementary Figures

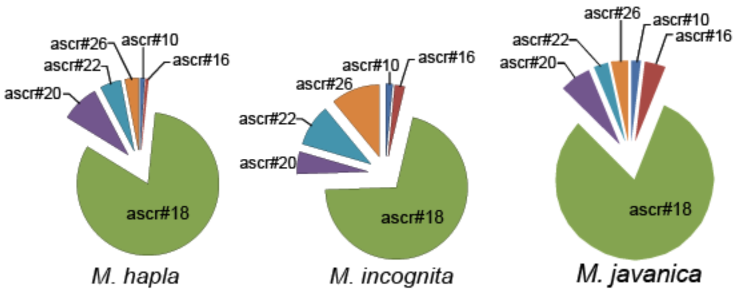

Supplementary Figure 1: Relative abundances of identified ascarosides, as determined from integration of HPLC-MS ion chromatograms, in the *exo*-metabolomes of three *Meloidogyne* species. All three species also produce trace amounts of ascr#24 (not shown).

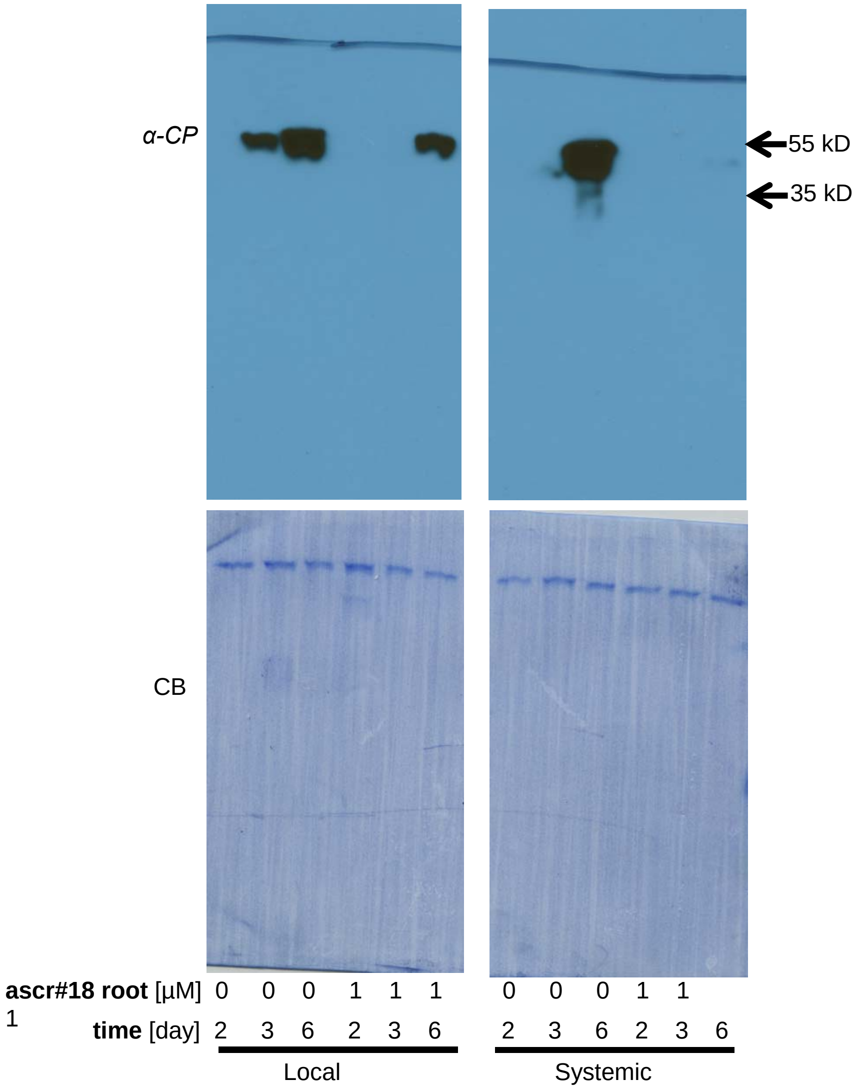

Supplementary Figure 2: Ascr#18 enhanced resistance to Turnip Crinkle Virus (TCV). Quantification of TCV coat protein (CP) in inoculated (local) and uninoculated (systemic) leaves of plants root-pretreated for 24 h with ascr#18. Leaves were harvested at 2, 3, and 6 dpi for immunoblot analysis with the anti-CP antibody. Coomassie blue staining (CB) served as loading control.

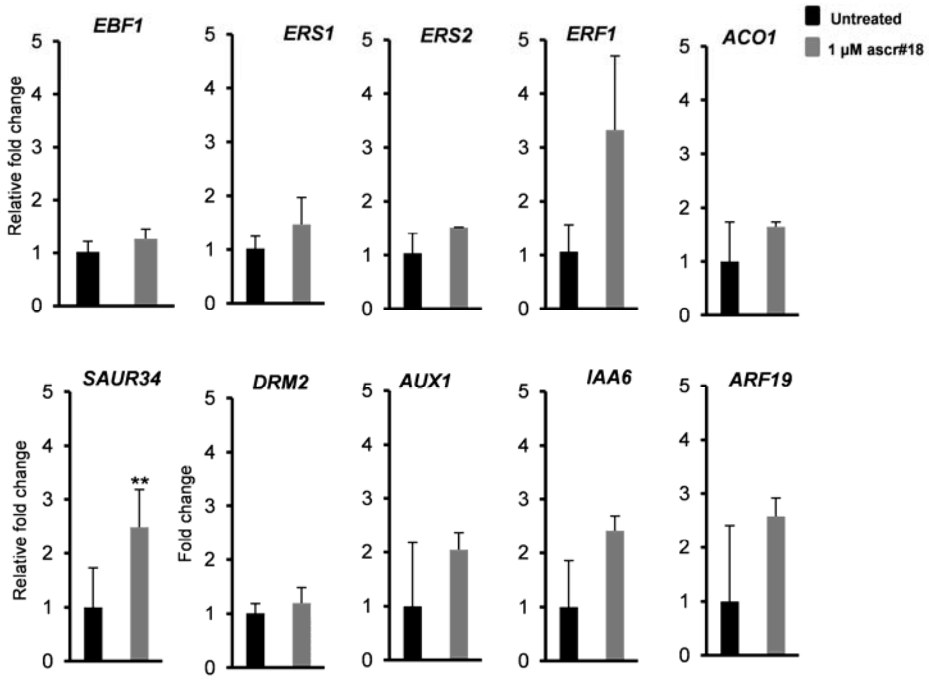

Supplementary Figure 3: Ascr#18 enhances expression of defense-related genes in *Arabidopsis* leaves. Transcript levels as measured by qRT-PCR of ethylene- and auxin-responsive gene markers in leaves from plants root-pretreated with ascr#18 (1 μM). Gene transcript levels of *EBF1, ERS1, ERS2, ERF1, ACO1, SAUR34, DRM2, AUX1, IAA6 and ARF19* were determined at 24 h post pretreatment (hpt). **P ≤ 0.005, two-tailed *t*-test.

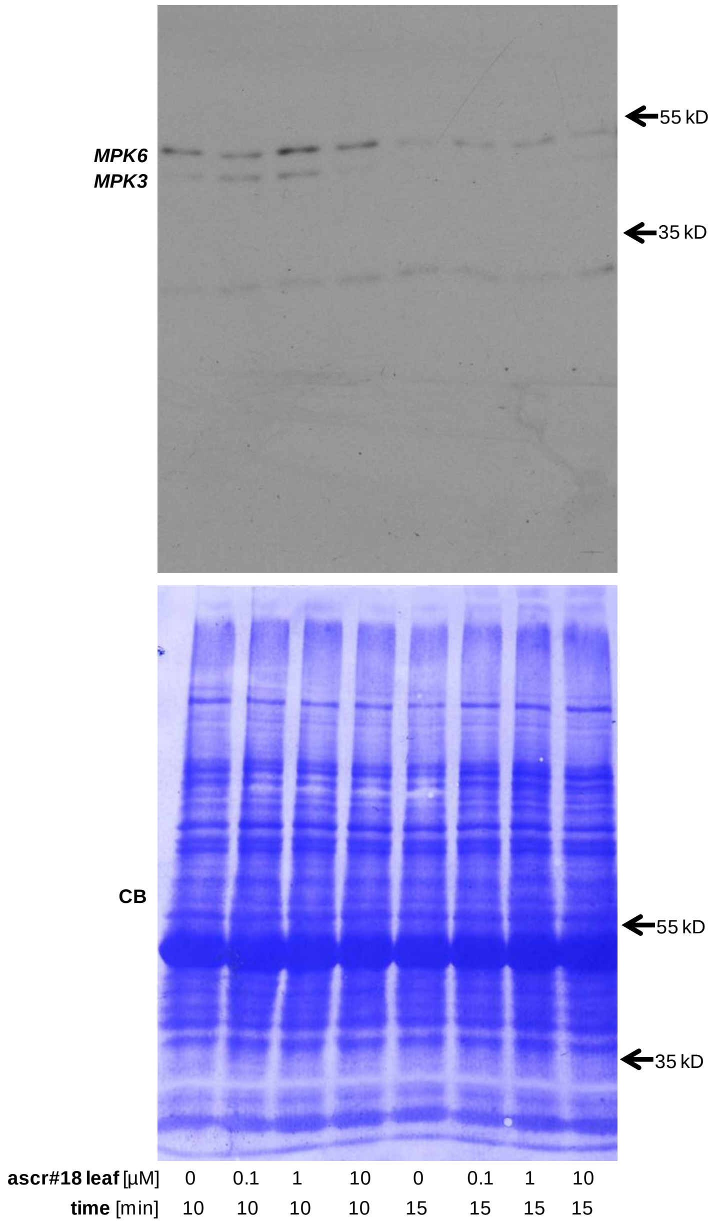

Supplementary Figure 4: Ascr#18 activates MAPKs in *Arabidopsis* leaves. Activation of MPK3 and MPK6 in *Arabidopsis* 10 and 15 min after leaf pretreatment with ascr#18. CB served as loading control.

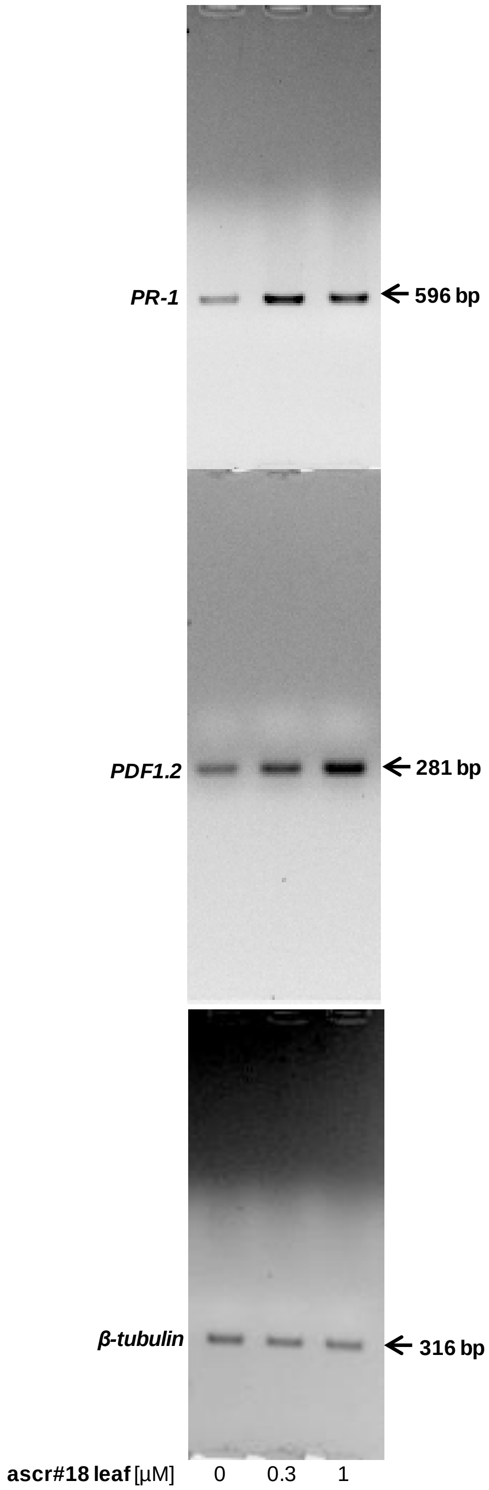

Supplementary Figure 5: Ascr#18 induces SA and JA marker genes in *Arabidopsis* leaves. Induction of SA and JA marker genes *PR-1* and *PDF1.2* respectively, after syringe infiltration of *Arabidopsis* leaves with ascr#18, as measured by qRT-PCR. *β-tubulin* was used as internal control.

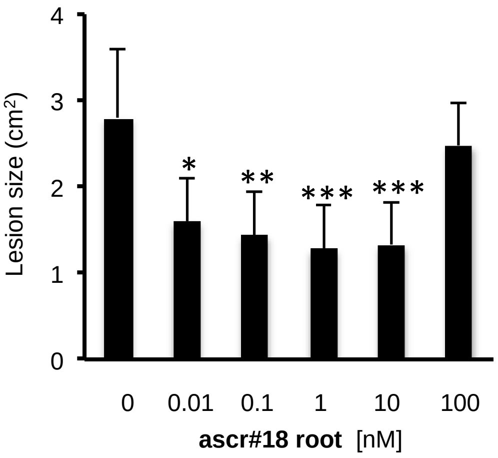

Supplementary Figure 6: Ascr#18 enhances disease resistance in tomato. Tomato cv. M82 plants were root-pretreated with the indicated concentrations of ascr#18 for 48 h before inoculation with *P. infestans* (US22) (4000 sporangia/mL) using a detached leaflet assay. Four leaves per plant from four plants were used for each concentration in a detached leaflet assay. Lesion size was determined at 5 dpi to assess disease symptoms. Data are average ± SD (n = 16, where n denotes the number of independent samples). *P ≤ 0.05; **P ≤ 0.005; ***P ≤ 0.0005, two-tailed *t*-test.

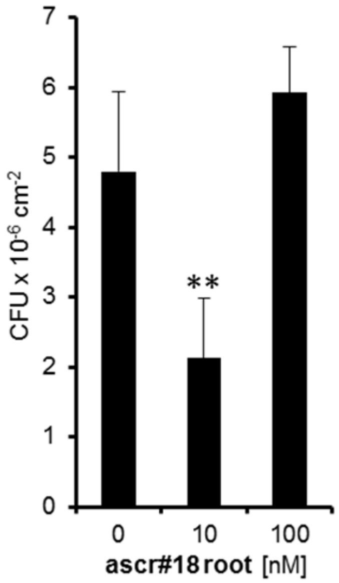

Supplementary Figure 7: Enhanced resistance to *Pst DC3000* in tomato cv. M82 root-pretreated with ascr#18 for 48 h. Bacterial growth was assayed at 4 dpi. Data are average ± SD (n = 6). **P ≤ 0.005, two-tailed *t*-test.

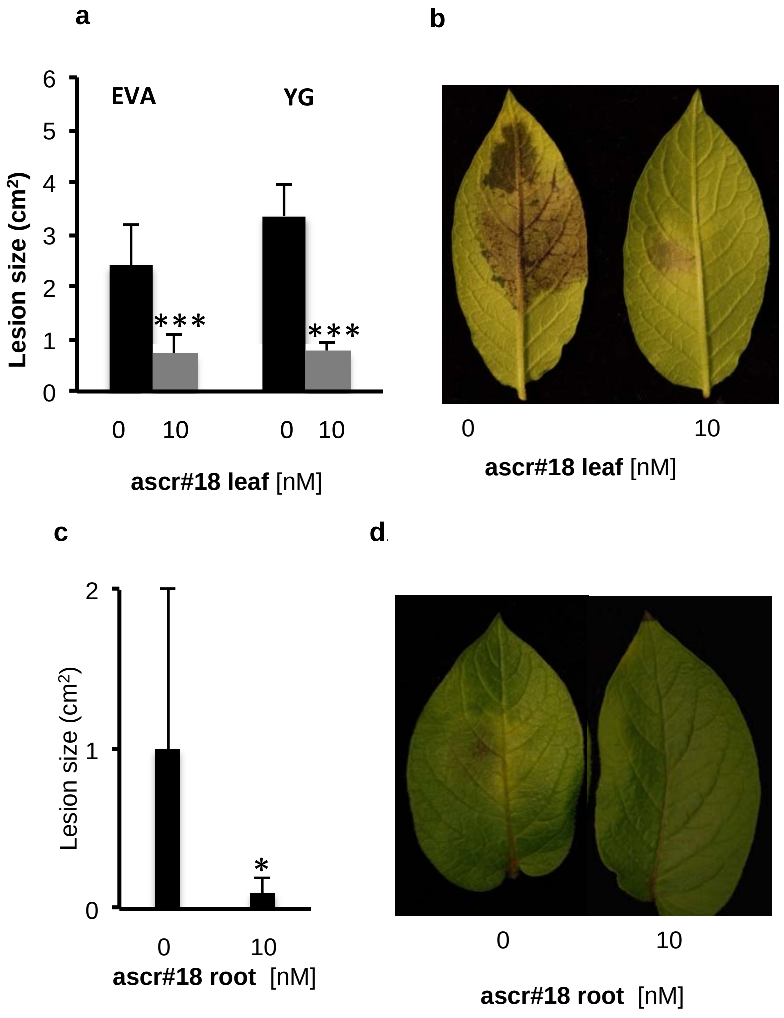

Supplementary Figure 8: Effect of ascr#18 on resistance to *P. infestans* in potato.

(a,b) Potato cultivars Eva and Yukon Gold (YG) were sprayed with the indicated ascr#18 concentrations 48 h before inoculation with *P. infestans* (US22) (4000 sporangia/mL). **(a)** Using a detached leaflet assay lesion size was determined at 5 dpi to assess disease symptoms. **(b)** Pictures of the blighted area in Yukon Gold were taken at 6 dpi. Data are average ± SD (n = 10, where n denotes the number of independent samples. *P ≤ 0.05; **P ≤ 0.005; ***P ≤ 0.0005, two-tailed *t*-test. **(c,d)** Potato cultivar Désirée was pretreated via root bathing using the indicated ascr#18 concentrations for 48 h before inoculation with *P. infestans* (US22) (4000 sporangia/mL). **(c)** Size of lesions in the detached leaflet assay caused by *P. infestans* was measured at 5 dpi. **(d)** Photographs of inoculated potato leaflets were taken at 5 dpi. Data are average ± SD (n = 7, where n denotes the number of independent samples). *P ≤ 0.05, two-tailed *t*-test.

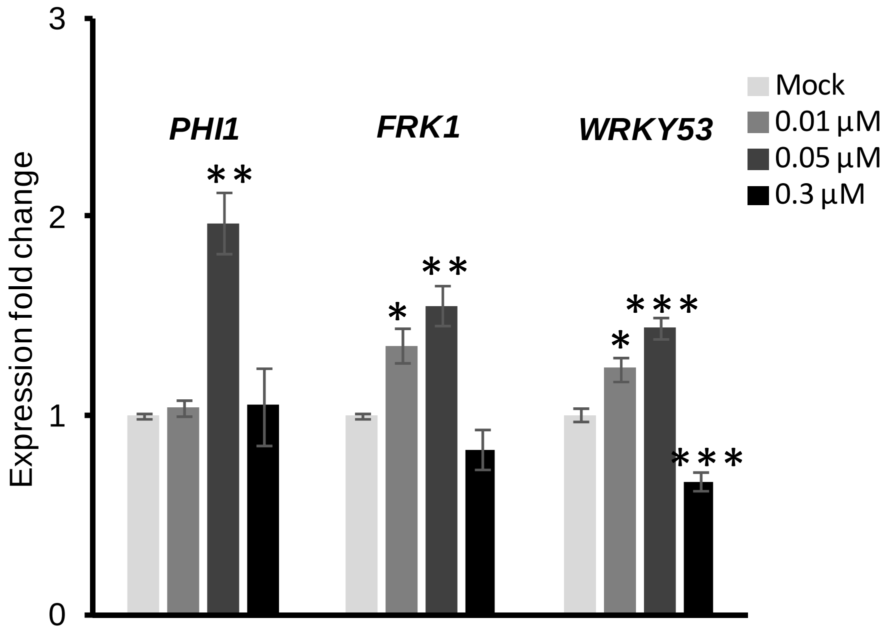

Supplementary Figure 9: Ascr#18 enhances expression of defense-related genes in *Arabidopsis* roots. Eight days old *Arabidopsis* seedlings were treated by root bathing using the indicated concentrations of ascr#18. Root samples were collected 6 h after treatment for RNA extraction. Transcript levels of *PHI1*, *FRK1* and *WRKY53* were determined by qRT-PCR and normalized to endogenous *UFP* (AT4G01000). $^*P \leq 0.05$; $^{**}P \leq 0.005$; $^{***}P \leq 0.0005$, two-tailed *t*-test.

Supplementary Tables

Supplementary Table 1: Chemical structures and high-resolution mass spectroscopic data, acquired in negative-ion electrospray ionization mode, of ascarosides detected in plant-parasitic nematodes. x: present; o: not detected. SMID: Small Molecule IDentifier, see www.smid-db.org for details on nomenclature.

SMID	n[a]	*m/z* [M-H]$^-$, calculated	*m/z* [M-H]$^-$, observed	***M. hapla***	***M. incognita***	***M. javanica***	***H. glycines***	***P. brachyurus***
ascr#10	6	303.1808	303.1813	x	x	x	o	o
ascr#16	7	317.1964	317.1959	x	x	x	o	o
ascr#18	8	331.2121	331.2129	x	x	x	x	x
ascr#20	9	345.2277	345.2267	x	x	x	o	o
ascr#22	10	359.2434	359.2429	x	x	x	o	o
ascr#24	11	373.2596	373.2653	x	x	x	o	o
ascr#26	12	387.2752	287.2727	x	x	x	o	o

[a]n: number of CH_2 groups in the side chains of the ascarosides (see graphic below)

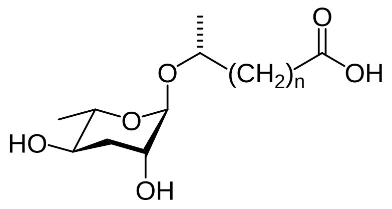

Supplementary Table 2: Induction of plant immune responses with various ascarosides. Leaf pretreatment by syringe infiltration of different ascaroside concentrations (0.01, 0.3, and 10 μM) were used for *PR-1*/*PDF1.2* expression studies in *Arabidopsis*. Resistance to *P. infestans* in tomato (M82) was performed using root pretreatment with 0.01 or 1 μM ascaroside. (+) indicates enhanced expression or resistance while (-) indicates no effect.

	ascr#18	ascr#3	ascr#9	oscr#9
PR-1 expression in Arabidopsis	+	+	+	±
PDF1.2 expression in Arabidopsis	+	+	+	-
P. infestans resistance in tomato	+	-	+	-

Supplementary Table 3: Primers used in this study.

Primer Name	Sequence
AtPR-1 F	TCGTCTTTGTAGCTCTTGTAGGTG
AtPR-1 R	TAGATTCTCGTAATCTCAGCTCT
AtPDF1.2-F	TCATGGCTAAGTTTGCTTCC
AtPDF1.2-R	AATACACACGATTAGCACC
AtTubulin-S	GTCCAGTGTCTGTGATATTGCACC
AtTubulin-R	TTACGAATCCGAGGGAGCCATTG
HvPR1b-F	GGACTACGACTACGGCTCCA
HvPR1b-R	GGCTCGTAGTTGCAGGTGAT
HvUbiquitin-F	ACCCTC GCCGACTACAACAT
HvUbiquitin-F	CAGTAGTGGCGGTCGAAGTG
AtFRK1-fw	TGCAGCGCAAGGACTAGAG
AtFRK1-rv	ATCTTCGCTTGGAGCTTCTC
AtPHI-fw	TTGGTTTAGACGGGATGGTG
AtPHI-rv	ACTCCAGTACAAGCCGATCC
qAtUBQ-fw	GGCCTTGTATAATCCCTGATGAATAAG
qAtUBQ-rv	AAAGAGATAACAGGAACGGAAACATAG
AtPR4-F	CTGGACCGCCTTCTGCGGG
AtPR4-R	AGCCTCCGTTGCTGCATTGGT
AtAOS-F	TCTTCTCTTCGCCACGTGC
AtAOS-R	GGTTATGAACTTGATGACCCGC
AtLOX2-F	TTGCTCGCCAGACACTTGC
AtLOX2-R	GGGATCACCATAAACGGCC
AtGSTF6-F	GGCAGGAATCAAAGTTTTCG
ATGSTF6-R	CGACCAAAGTGAAGTGGTCA
LeGST-F	GCCCTTCCATCTTGCCTAAAG
LeGST-R	GTCACCAAAACAACTCCAGATGC
Leβ-1,3-glucanase-F	TGCTACATACTCGGCCCTTGAA
Leβ-1,3-glucanase-R	TTTGGCTGCCTGTTTGGTGT
LeGRAS4	CCGTCCTGATTTATTCATCCAT
LeGRAS4	TCGTGTGACGAAAAATGGAGT
LeActin	GAGCGTGGTTACTCGTTCA
LeActin	GAGCGTGGTTACTCGTTCA
LeAOS2-F	GCAACGAAGGATCCGAAAAT
LeAOS2-R	ACTGGCCGATAGTGACAGTG
qEBF1-F	CAGATCTTTAGTTTTGCCGGTGA
qEBF1-R	AGCATCCTTTGGGTTTGGGT
qERS1-F	CTGATTCTGTCTGCAGA

qERS1-R	TGTGTGAATTCCACACCCTGTG
qERS2-F	GCCAAAACATTGTAAAGTATATGCA
qERS2-F	CTTCCTGACGTCAATGATCAGT
qERF1-F	TTTCTCGATGAGAGGGTC
qERF1-R	AAGCTCCTCAAGGTACTG
qACO1-F	AGGAACTCAGCAAGACGATGG
qACO1-R	GACGTGGGCATTCTGGGTAT
qSAUR34-F	CGACAGTTCCAAGAGGGCAT
qSAUR34-R	GTTCAAACCCGTAAACCCGC
qDRM2-F	CCCTTGACATCAAAGGTGTAGGA
qDRM2-R	GGGTGAGAAGGCTTGTCGAA
qAUX1-F	GATGAGATAAGCAGTCCAGCTTCC
qAUX1-R	CAGCTGCGCATCTAACCAAGTG
qIAA6-F	TTCGATTGGGTCTTCCAGGAGATA
qIAA6-R	ATCTTGCTGGAGACCAAAACCA
qARF19-F	CCTCCTGTGGGAAGTCTTGTGGTTTAC
qARF19-R	GCTCCAACCTGTGGTAAGCAAGTG
qWRKY53-F	TCACTTTTTCTGACCACTTTGG
qWRKY53-R	AAGGAAGAGATATGTTAAGTTGGG

Supplementary Methods

Synthesis of ascr#18.

General synthetic procedures. Starting materials were synthesized as described in cited references or purchased from Sigma-Aldrich or Acros Organics and used without further purification. Anhydrous solvents were prepared with 4 Å molecular sieves. NMR spectra were recorded on a Varian INOVA-600 (600 MHz for ^{1}H, 151 MHz for ^{13}C), INOVA-500 (500 MHz for ^{1}H and 125 MHz for ^{13}C), and INOVA-400 (400 MHz for ^{1}H, 100 MHz for ^{13}C) instruments. Flash chromatography was performed using a Teledyne ISCO CombiFlash system.

Step 1. (9*R*)-hydroxydec-1-ene

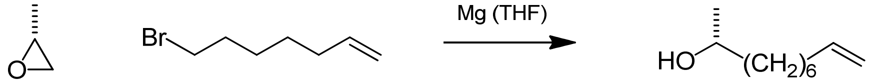

A solution of 7-bromoheptene (300 µg, 2 mmol) in dry THF (1 mL) was added drop wise to magnesium (240 mg, activated with iodine) in THF (500 µL). After stirring at RT for 1 h the Grignard solution was transferred, cooled to -40°C and treated with CuI (30 mg, 158 µmol). After stirring for 1 min (*R*)-propylene oxide (100 µL, 2 mmol) in THF (500 µL) was added and the solution stirred for 1.5 h. The reaction was quenched with NH_4Cl (1 mL), extracted with diethyl ether, dried over Na_2SO_4, and concentrated in vacuum. Flash column chromatography on silica gel using an ethyl acetate – hexane gradient (0 to 20%) afforded (8*R*)-hydroxydec-1-ene (56 mg, 359 µmol, 18% yield) as a colorless liquid.

^{1}H NMR (600 MHz, chloroform-d): δ 1.18 (3H, d, *J* = 6.2 Hz), 1.25-1.50 (10H, m), 2.01-2.07 (2H, m), 3.76-3.82 (1H, m), 4.91-4.95 (1H, m), 4.97-5.01 (1H, m), 5.81 (1H, ddt, *J* = 17.1 Hz, 10.4 Hz, 6.7 Hz). NMR spectroscopic data are in agreement with those reported in[1].

Step 2. (9*R*)-(3'*R*,5'*R*-dibenzoyloxy-6'*S*-methyl-(2*H*)-tetrahydropyran-2-yloxy)-dec-1-ene

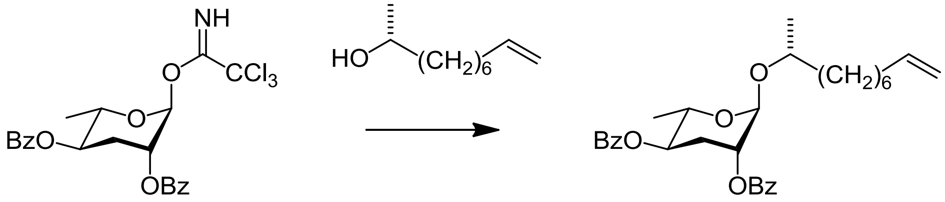

A solution of 2,4-di-*O*-benzoylascarylose[2] (139 mg, 390 µmol) in dry DCM (3 mL) was treated with trichloroacetonitrile (84 µL) and DBU (5 µL). After stirring at RT for 30 min the solution was concentrated in vacuum. Flash column chromatography on silica gel using a mixture of ethyl acetate in hexane (20%) afforded (3'*R*,5'*R*-dibenzoyloxy-6'*S*-methyl-(2*H*)-tetrahydropyran-2-yloxy)-1-(2,2,2-trichloroacetimide) (152 mg, 302 µmol, 78%) as a colorless oil. A solution of 2,4-di-*O*-benzoyl-ascarylose-1-(2,2,2-trichloroacetimide) (152 mg, 302 µmol) in dry DCM (3 mL) at 0 °C was treated with (9*R*)-hydroxydec-1-ene (55 mg, 350 µmol) and trimethylsilyloxy triflate (5 µL). After 3 h the solution was washed with saturated aqueous $NaHCO_3$ solution (0.5 mL), dried over Na_2SO_4 and concentrated in vacuum. Flash column chromatography on silica gel using a ethyl acetate – hexane gradient (5 to 20%) afforded (9*R*)-(3'*R*,5'*R*-dibenzoyloxy-6'*S*-methyl-(2*H*)-tetrahydropyran-2-yloxy)-dec-1-ene (91.1 mg, 184 µmol, 61%) as a colorless oil.

^{1}H NMR (400 MHz, chloroform-d): δ 1.20 (3H, d, *J* = 6.1 Hz), 1.30 (3H, d, *J* = 6.1 Hz), 1.33 – 1.72 (10H, m), 2.09 (2H, m), 2.23 (1H, ddd, *J* = 13.5 Hz, *J* = 11.4 Hz, *J* = 2.9 Hz), 2.44 (1H, m), 3.87 (1H, m), 4.15 (1H, dq, *J* = 9.8 Hz, *J* = 6.1 Hz), 4.95 (1H, ddt, *J* = 10.2 Hz, *J* = 2.2 Hz, *J* = 1.3 Hz), 4.98 (1H, s.br), 5.02 (1H, ddt, *J* = 17.1, Hz. *J* = 2.2 Hz, *J* = 1.6 Hz), 5.17 (1H, s.br), 5.21 (1H, ddd, *J* = 10.3 Hz, *J* = 4.6 Hz), 5.83 (1H, ddt, *J* = 17.1 Hz, *J* = 10.3 Hz, *J* = 6.8 Hz), 7.45-7.51 (4H, m), 7.57-7.62 (2H, m), 8.06 (2H, m), 8.13 (2H, m); ^{13}C NMR (100 MHz, chloroform-d): δ 17.84, 19.14, 25.65, 28.84, 29.08, 29.38, 29.68, 33.76, 37.08, 66.89, 70.62, 71.21, 72.53, 93.72, 114.20, 128.38, 129.55, 129.80, 129.82, 129.96, 133.12, 133.17, 139.01, 165.59, 165.72. High-resolution MS data for this compound have been reported previously, see[3].

Step 3. Ethyl (10*R*)-(3'*R*,5'*R*-dibenzoyloxy-6'*S*-methyl-(*2H*)-tetrahydropyran-2-yloxy)-undec-2-enoate

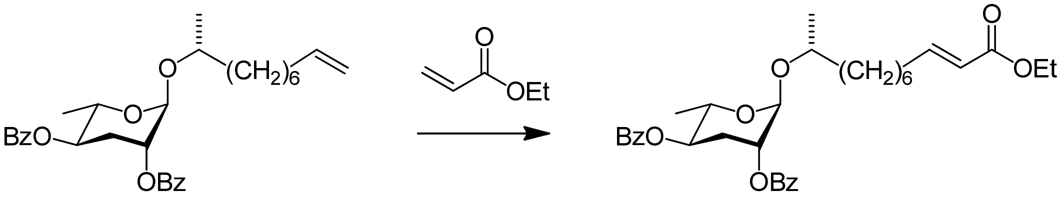

A solution of (9*R*)-(3'*R*,5'*R*-dibenzoyloxy-6'*S*-methyl-(*2H*)-tetrahydropyran-2-yloxy)-dec-1-ene (62 mg, 125 μmol) and ethyl propenoate (66 mg, 626 μmol) in DCM (5 mL) was treated with 1.4-benzoquinone (1.4 mg, 13 μmol) and Grubbs-II catalyst (5.3 mg, 6.3 μmol). After stirring at 40 °C for 15 h, the reaction was filtered through a pad of silica using DCM: ethyl acetate (3:1). Flash column chromatography on silica gel using a ethyl acetate – hexanes gradient (10 to 50%) afforded ethyl (10*R*)-(3'*R*,5'*R*-dibenzoyloxy-6'*S*-methyl-(*2H*)-tetrahydropyran-2-yloxy)-undec-2-enoate (55 mg, 97 μmol, 78%) as a colorless oil.

^{1}H NMR (400 MHz, chloroform-d): δ 1.19 (3H, d, *J* = 6.1 Hz), 1.27 (3H, t, *J* = 7.1 Hz), 1.28 (3H, d, *J* = 6.3 Hz), 1.33-1.70 (10H, m), 2.16-2.26 (3H, m), 2.38-2.46, (1H, m), 3.84 (1H, m), 4.07-4.15 (1H, m), 4.17 (2H, q, *J* = 7.1 Hz), 4.95 (1H, s.br), 5.12-5.23 (2H, m), 5.78-5.85 (1H. m), 6.97 (1H, dt, *J* = 15.6 Hz, 7.0 Hz), 7.42-7.50 (4H, m), 7.55-7.62 (2H, m), 8.01-8.06 (2H, m), 8.09-8.14 (2H, m). ^{13}C NMR (100 MHz, chloroform-d): δ 14.42, 18.03, 19.30, 25.78, 28.16, 29.28, 29.53, 29.87, 32.32, 37.23, 60.29, 67.09, 70.80, 71.38, 72.78, 93.93, 117.65, 121.44, 128.58, 129.73, 129.98, 129.99, 130.13, 133.32, 133.38, 149.44, 165.80, 165.93, 166.89.

Step 4. (10*R*)-(3'*R*,5'*R*-dihydroxy-6'*S*-methyl-(*2H*)-tetrahydropyran-2-yloxy)-undec-2-enoic acid (ascr#17)

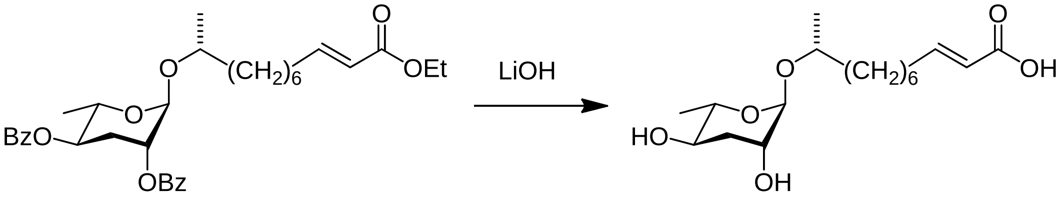

A solution of ethyl (10*R*)-(3'*R*,5'*R*-dibenzoyloxy-6'*S*-methyl-(*2H*)-tetrahydropyran-2-yloxy)-undec-2-enoate (55 mg, 97 μmol) in THF (1 mL) was added to a solution of lithium hydroxide (48 mg, 2 mmol) in water (380 μL) and 1,4-dioxane (2 mL). After stirring at 67 °C for 3 h the mixture was neutralized with acetic acid and concentrated in vacuum. Flash column

chromatography on silica gel using a methanol – dichloromethane gradient (0 to 30%) afforded (10*R*)-(3'*R*,5'*R*-dihydroxy-6'*S*-methyl-(*2H*)-tetrahydropyran-2-yloxy)-undec-2-enoic acid (ascr#17) (25.2 mg, 76.4 µmol, 79%) as a colorless oil.

^{1}H NMR (500 MHz, methanol-d$_4$): δ 1.12 (3H, d, *J* = 6.1 Hz), 1.21 (3H, d, *J* = 6.3 Hz), 1.33 – 1.60 (10H, m), 1.76 (1H, ddd, *J* = 13.3 Hz, *J* = 11.4 Hz, *J* = 3.1 Hz), 1.95 (1H, dt.br, *J* = 13.1 Hz, *J* = 4.1 Hz), 2.23 (2H, ddt, *J* = 7.3 Hz, *J* = 1.7 Hz, *J* = 7.6 Hz), 3.52 (1H, ddd, *J* = 11.3 Hz, *J* = 9.5 Hz, *J* = 4.6 Hz), 3.63 (1H, dq, *J* = 9.3 Hz, *J* = 6.4 Hz), 3.71 (1H, m), 3.78 (1H, m), 4.64 (1H, s.br), 5.80 (1H, dt, *J* = 15.7 Hz, *J* = 1.4 Hz), 6.95 (1H, dt, *J* = 15.6 Hz, *J* = 7.0 Hz); ^{13}C NMR (100 MHz, methanol-d$_4$): δ 18.27, 19.53, 26.95, 29.40, 30.40, 30.61, 33.29, 36.09, 38.51, 68.45, 70.10, 71.30, 72.62, 97.67, 122.75, 151.25, 170.37. High-resolution MS data for this compound have been reported previously, see[3].

Step 5. (10*R*)-(3'*R*,5'*R*-dihydroxy-6'*S*-methyl-(*2H*)-tetrahydropyran-2-yloxy)-undecanoic acid (ascr#18)

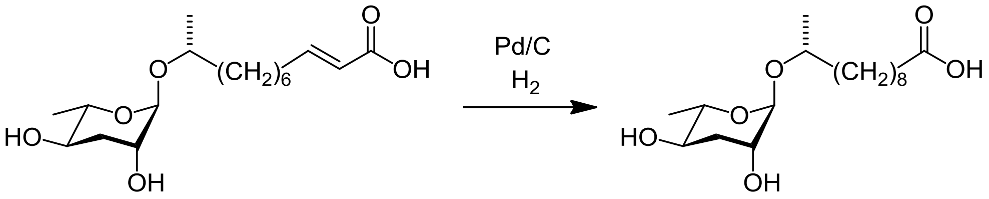

A solution of (10*R*)-(3'*R*,5'*R*-dihydroxy-6'*S*-methyl-(*2H*)-tetrahydropyran-2-yloxy)-undec-2-enoic acid (5 mg, 104 µmol) in methanol (1 mL) was treated with Pd/C (10% w/w) and hydrogenated for 14 h. The solution was filtered and concentrated in vacuum to afford (10*R*)-(3'*R*,5'*R*-dihydroxy-6'*S*-methyl-(*2H*)-tetrahydropyran-2-yloxy)-undecanoic acid (4.4 mg, 76.4 µmol, 73%) as a colorless oil.

^{1}H NMR (500 MHz, methanol-d$_4$): δ 1.12 (H, d, *J* = 6.1 Hz), 1.21 (3H, d, *J* = 6.3 Hz), 1.33 – 1.60 (14H, m), 1.76 (1H, ddd, *J* = 13.3 Hz, *J* = 11.4 Hz, *J* = 3.1 Hz), 1.95 (1H, dt.br, *J* = 13.1 Hz, *J* = 4.1 Hz), 2.27 (2H, t, *J* = 7.6 Hz), 3.52 (1H, ddd, *J* = 11.3 Hz, *J* = 9.5 Hz, *J* = 4.6 Hz), 3.63 (1H, dq, *J* = 9.3 Hz, *J* = 6.4 Hz), 3.71 (1H, m), 3.78 (1H, m), 4.64 (1H, s.br); ^{13}C NMR (100 MHz, methanol-d$_4$): δ 18.11, 19.37, 26.40, 26.88, 30.37, 30.48, 30.61, 30.67, 35.97, 38.42, 68.34, 69.99, 71.17, 72.51, 97.56, 178.6. High-resolution MS data for this compound have been reported previously, see[3].

Supplementary References

1 Trollsås, M. *et al.* Preparation of a Novel Cross-Linked Polymer for Second-Order Nonlinear Optics. *J. Am. Chem. Soc.* **118**, 8542-8548 (1996).

2 Jeong, P. Y. *et al.* Chemical structure and biological activity of the Caenorhabditis elegans dauer-inducing pheromone. *Nature* **433**, 541-545 (2005).

3 von Reuss, S. H. *et al.* Comparative metabolomics reveals biogenesis of ascarosides, a modular library of small molecule signals in *C. elegans*. *J. Am. Chem. Soc.* **134**, 1817–1824 (2012).
